# Clinical and Genetic Characteristics, Management and Long-Term Follow-Up of Turkish Patients with Congenital Hyperinsulinism

**DOI:** 10.4274/jcrpe.2408

**Published:** 2016-06-06

**Authors:** Ayla Güven, Ayşe Nurcan Cebeci, Sian Ellard, Sarah E. Flanagan

**Affiliations:** 1 Göztepe Training and Research Hospital, Clinic of Pediatric Endocrinology, İstanbul, Turkey; 2 Amasya University Faculty of Medicine, Department of Pediatrics, Amasya, Turkey; 3 Derince Training and Research Hospital, Clinic of Pediatric Endocrinology, Kocaeli, Turkey; 4 University of Exeter Medical School, Institute of Biomedical and Clinical Science, Exeter, United Kingdom

**Keywords:** Hyperinsulinism, pancreatectomy, diabetes mellitus, ATP-sensitive potassium (KATP) channel

## Abstract

**Objective:**

Mutations in the KATP channel genes is the most common cause of congenital hyperinsulinism (CHI) of infancy. Our aim was to report the clinical and genetic characteristics, treatment modalities, and long-term prognosis of patients with CHI.

**Methods:**

Clinical and biochemical findings, operation procedures, and results of genetic analysis were retrospectively evaluated in 22 CHI patients from two pediatric endocrine centers in Turkey.

**Results:**

Seven of the patients were born large for gestational age. Hypoglycemia was diagnosed within the first 24 hours of life in 9 patients and treatment with diazoxide (n=21) and/or somatostatin (n=8) had been attempted. Seven patients (31.8%) were unresponsive to medical treatment and underwent pancreatectomy. Histological examination of the pancreas confirmed diffuse disease in 6 patients. Diabetes developed in 3 patients following pancreatectomy (10 years, 2.5 years, and immediately after operation). The remaining four patients had neither recurrence of CHI nor of diabetes during the 3.67±0.7 years of follow-up. Sequence analysis identified mutations in 12 out of 19 patients (63%). Mutations in the ABCC8 gene were the most common finding and were found in 6 out of 7 patients who underwent pancreatectomy. Other mutations included a paternally inherited KCNJ11 mutation, a homozygous HADH mutation, and a heterozygous GLUD1 mutation.

**Conclusion:**

Mutations in the ABCC8 gene were the most common cause of CHI in our cohort. These mutations were identified in 85% of patients who underwent pancreatectomy. The development of diabetes mellitus after pancreatectomy may occur at any age and these patients should be screened regularly.

**WHAT IS ALREADY KNOWN ON THIS TOPIC?**

There is no follow-up study in patients with congenital hyperinsulinism (CHI) in Turkey.

**WHAT THIS STUDY ADDS?**

This study is the longest follow-up in patients with CHI in Turkey.

## INTRODUCTION

Congenital hyperinsulinism (CHI) is the most common cause of severe, persistent or recurrent hypoglycemia in the neonatal period and infancy. Mutations in the ABCC8 and KCNJ11 genes encoding the ATP-sensitive potassium (KATP) channel, which regulates the insulin secretion from pancreatic beta cell, are the leading cause of congenital hyperinsulinism. Rarely, mutations in the genes which encode glucokinase (GCK), glutamate dehydrogenase (GLUD1), 3-hydroxyacyl-coenzyme A dehydrogenase (HADH), hepatocyte nuclear factor 4 (HNF4A), hepatocyte nuclear factor 1 (HNF1A), monocarboxylate transporter 1 (SLC16A1), and the mitochondrial inner membrane protein UCP2 (UCP2) have been reported to cause hyperinsulinemic hypoglycemia (1).

Clinical signs and symptoms can present at any age from the neonatal period to adulthood and may vary depending on the specific mutation identified (2). Since recurrent severe hypoglycemia has a negative effect on neurocognitive function, especially during the first years of life, early recognition and treatment of this condition can be expected to provide a favorable prognosis. In patients who do not respond to medical therapy, pancreatectomy should be considered. The differentiation between focal and diffuse CHI before surgery would affect the success of surgical treatment (3).

Pancreatectomy is usually performed by removing 95-98% of the pancreatic tissue. This procedure comes with a risk of the patient developing diabetes mellitus and exocrine pancreatic insufficiency. Only a few studies have reported the long-term outcomes of patients following pancreatectomy (4,5,6,7,8,9,10,11).

In this study, we report the clinical features, treatment modalities, and long-term follow-up of our patients with CHI. We aim to contribute further information to the literature by demonstrating the clinical and mutational analyses of all patients at admission and also report the outcomes of patients who underwent pancreatectomy.

## METHODS

We retrospectively reviewed the medical records of patients diagnosed with CHI at the Göztepe Training and Research Hospital Pediatric Endocrinology Clinic, İstanbul (Centre 1) and at the Derince Training and Research Hospital Pediatric Endocrinology Clinic, Kocaeli (Centre 2).

We identified 24 patients with CHI (Centre 1, n=20; Centre 2, n=4). Two patients were excluded from the study since they were clinically diagnosed as Beckwith-Wiedemann syndrome. Twenty-two patients (7 females, 15 males) were recruited, and clinical data were extracted from the patient files. The diagnosis of CHI was based on detectable insulin levels during spontaneous or provoked hypoglycemia. A fasting provocation test was undertaken in two patients who had a history of hypoglycemia after overnight fasting and in another patient who was 171/12 years old. This patient was diagnosed with hypoglycemia at the age of one year, diazoxide treatment was initiated at the age of 3 years, but the patient had missed follow- up for 14 years. He had mental retardation due to recurrent hypoglycemic attacks because of poor compliance with treatment. He suffered from a hypoglycemic attack on admission to our clinic. As severe hypoglycemia was noted during the fasting provocation test, diazoxide therapy was restarted and he did not have any further hypoglycemic attacks (case 10).

For all patients who underwent fasting provocation, the test was stopped when the blood glucose level fell below 45 mg/dL (12) and a blood sample was drawn for measurements of glucose, insulin, ketones, cortisol, growth hormone, ammonia, lactate, and pyruvate. Glucagon was subsequently administered at a dose of 30 μg/kg (max 1 mg) subcutaneously or intramuscularly, and blood glucose was re-measured thirty minutes later.

In case 1, with a history of protein sensitivity, a leucine provocation test was performed to confirm the diagnosis.

All patients were initially treated with intravenous high dose (10-15 mg/kg per min) glucose infusion and diazoxide (5-20 mg/kg per day). With the patient receiving a normal diet and following a 8-12 hr fast, absence of hypoglycemia (>55 mg/dL) with diazoxide treatment in a dose <15 mg/kg/d indicates responsiveness to diazoxide. The patients in whom the hypoglycemia (<55 mg/dL) persisted, despite receiving a maximum diazoxide dose (20 mg/kg/day) for 48 hours, were considered as unresponsive.

In 8 patients who were unresponsive to diazoxide, treatment with octreotide (5-40 mg/kg per day) was initiated. Two patients who underwent pancreatectomy received nifedipine (0.25-2.5 mg/kg per day) prior to pancreatectomy, but nifedipine therapy failed to prevent hypoglycemic episodes.

In case 4, a positron emission tomography scan using fluorine 18 L-3,4- dihydroxyphenylalanine (18F-DOPA PET) was performed in Frankfurt, Germany. Focal involvement was not detected. As 18F-DOPA PET-CT scanning is not available in Turkey, scans were not performed on other patients in our cohort.

In four patients who did not respond to diazoxide and octreotide therapy, 98% pancreatectomy was performed in our hospital. Patients 7, 11, and 12 were operated in other hospitals and subsequently referred to our clinic.

### Genetic Analysis

Genetic testing was performed by the University of Exeter Medical School with written informed consent obtained from parents of all patients. Genomic DNA was extracted from peripheral leukocytes of 19 patients using standard procedures and the single exon of KCNJ11 and 39 exons of ABCC8 were amplified by polymerase chain reaction (primers available on request). The amplicons were sequenced using Big Dye Terminator cycler sequencing Kit v3.1 (Applied Biosystems, Warrington, UK). Sequencing reactions were analyzed on an ABI3730 (Applied Biosystems, Warrington, UK) and compared to the reference sequences using Mutation Surveyor software (SoftGenetics, Pa., USA).

The GLUD1 gene was sequenced in one patient with hyperinsulinism and hyperammonaemia using previously reported methods (13). For all other patients without an ABCC8 or KCNJ11 mutation, sequencing analysis of the HADH gene was undertaken (14,15). If no HADH mutation was identified, HNF4A was sequenced in any patient diagnosed with CHI within the first 2 weeks of life (16).

When a mutation was identified and samples were available, the unaffected parents were tested to investigate their carrier status. Microsatellite analysis was undertaken on DNA extracted from the resected pancreatic tissue of one patient to investigate loss of maternal heterozygosity at chromosome 11p15.1.

### Statistical Analysis

Statistical analysis was performed using Statistical Package for the Social Sciences 18.0 (SPSS, Chicago, IL, USA) software. Shapiro-Wilk test was used to test the normality of the data. Descriptive data were expressed as mean ± standard deviation values. Skewed data were shown as median and interquartile range (IQR). Spearman’s correlation method was used in correlation analysis. For all tests, a p-value of less than 0.05 was accepted as statistically significant.

## RESULTS

The clinical characteristics of the patients with CHI are given in Table 1.

Seven patients (33.3%) were born large for gestational age (LGA). Seventeen patients were born at term. Cases 5 and 6 were siblings and 8 parents were known to be consanguineous.

The initial hypoglycemic episode was observed in the first 24 hours of life in nine patients (42.8%), and in the first week of life in seven patients (33.3%).

Thirteen patients (59%) were diazoxide-responsive. Diazoxide treatment was stopped between 15 days and 12 years in seven of these patients (53.8%) and no recurrence of hypoglycemia was observed. There was a history of birth asphyxia in the patient whose diazoxide therapy was stopped after 15 days. In this patient, the insulin level during hypoglycemia (30 mg/dL) was 40 mIU/mL and no genetic analysis was made.

After diazoxide therapy was discontinued, capillary blood glucose was checked before each feeding during minimum period of three days. During their hospitalization, we observed that babies could tolerate fasting for three hours, whereas toddlers and older children could tolerate a six-hour fasting period without hypoglycemia.

Octreotide was started in eight patients (36.3%) who did not respond to diazoxide. Of those, case 3 showed a good response. Overall, 63.6% of the patients were responsive to medical treatment and they did not have any hypoglycemic attacks during diazoxide and/or octreotide therapy with regular food intake. In one patient, normoglycemia was achieved through frequent feeds with a carbohydrate-rich diet.

Four patients who did not respond to medical treatment underwent pancreatectomy in our hospital at a median age of 0.43±0.32 years. Neither hypoglycemia nor hyperglycemia was observed in these patients following surgery. Diabetes did not develop during the 3.67±0.7 years of follow-up.

Case 4, at age 57/12 years, was found to have a HbA1c of %6.4, a fasting glucose level of 87 mg/dL, a fasting C- peptide level of 1.77 ng/mL, and a fasting insulin level of 1.63 mIU/mL. We wanted to perform an intravenous glucose tolerance test because the patient had vomited twice during oral glucose tolerance test. The parents did not consent to this procedure.

Cases 7, 11, and 12 underwent surgery in other centers at a mean age of 0.39±0.44 years. Hypoglycemia was observed in two of these three cases (cases 11 and 12) after subtotal pancreatectomy and they received nifedipine for two and three months, respectively. No hypoglycemic attacks were noted. Diabetes developed immediately after near-total pancreatectomy in case 7, and 2.5 years and 10 years after subtotal pancreatectomy in cases 11 and 12, respectively. All three patients are currently receiving intensive (basal and mealtime bolus) insulin treatment.

Histological examination of the resected pancreatic tissue confirmed diffuse disease in six patients, and a focal lesion was detected in the tail of the pancreas in one patient (case 4). The focal lesion could not be identified prior to the operation (18F DOPA-PET-CT scanning was not available).

### Genetic Analysis

Results of the genetic analyses are given in Table 2.

Mutations were detected in 12 out of the 19 patients (63.2%). Nine patients had ABCC8 mutation(s) and KCNJ11, GLUD1 and HADH mutation(s) were each identified in a single patient (Table 2). Three of the mutations were novel. The genetic results for the patient with the homozygous HADH mutation have been reported previously (14). Fourteen unaffected parents were heterozygous for a mutation and are therefore carriers of congenital hyperinsulinism.

A paternally inherited ABCC8 mutation was identified in the patient with a focal lesion (case 4). Analysis of nine microsatellite markers spanning chromosome 11p15.5 to 11p15.1 was performed using DNA extracted from the resected tissue, but no loss of heterozygosity was observed.

Case 3 was heterozygous for two novel KCNJ11 mutations. Family member testing identified both mutations in the unaffected father, confirming that the mutations were on the same allele (in cis). The patient responded well to octreotide treatment. At two years of age, he could tolerate overnight fasting under a very low dose octreotide (9 μg/kg/d). A clinical remission was considered, and octreotide treatment was gradually diminished and stopped. He is currently not requiring medication and is able to keep his glucose levels >70 mg/dL.

Consequently, mutations in the KATP channel gene were identified in ten patients. Of those, 4 patients had recessively inherited mutations and 85.5% underwent pancreatectomy. However, mutations in the KATP channel genes were not identified in all patients who underwent pancreatectomy.

In one patient with mildly elevated levels of ammonia (185 μg/dL, normal: 40-80), the genetic analysis identified a heterozygous missense mutation in the GLUD1 gene. This mutation was not detected in leukocyte DNA from the unaffected parents and it is therefore likely that the mutation was a de novo mutation.

The genetic analysis of seven patients who underwent pancreatectomy revealed mutation(s) in the ABCC8 gene in 6 patients. The mutation analysis results were available in only two patients prior to surgery. In one patient with diffuse pancreatic disease, the screening of the ABCC8, KCNJ11, HADH, and HNF4A genes did not identify a mutation. 

Birth weight was significantly higher in those patients with a ABCC8 gene mutation compared to those without an ABCC8 gene mutation (r=0.857, p=<0.001).

## DISCUSSION

In this study, we evaluated the clinical and genetic features and long-term follow-up outcomes in a group of Turkish patients with CHI. Severe hypoglycemia occurred in the first days of life in the majority of patients, and this was consistent with the findings of previous studies.

Mutations were identified in previously known CHI genes in more than half (63%) of patients. As in previous studies, the most common genetic etiology among diazoxide-unresponsive patients was ABCC8 mutations.

Snider et al (17), reported in their series of 417 cases that no mutations were found in 3.9% (11/282) of patients with diffuse disease who underwent pancreatectomy. Greer et al (18) did not identify ABCC8 or KCNJ11 mutations in 5 of 21 patients (23%) who underwent pancreatectomy. In another study reporting 175 patients with CHI, 13 of 70 patients (18%) who underwent pancreatectomy had no mutations in the ABCC8 or KCNJ11 genes (19). A more recent study indicated that 3% of patients with diffuse CHI had no mutations in the genes encoding the KATP channel (20). Similarly, we did not identify a mutation in one of our patients with diffuse disease who underwent pancreatectomy. It is possible that this patient has a large deletion and/or a non-coding mutation(s) which was not detected by Sanger sequencing.

Compound heterozygosity is not common in patients with CHI (17,20,21,22,23). Sogno Valin et al (21) identified compound heterozygous KATP channel mutations in 2 of 33 patients within their cohort. Of those, one patient responded to diazoxide treatment and the other underwent pancreatectomy. Arya et al (24) recently reported 45 patients with CHI who underwent near-total pancreatectomy. They found that one third of patients were compounded heterozygous. One patient in the present study (case 8) had a maternally inherited novel missense mutation and was unresponsive to diazoxide and octreotide therapy. A near-total pancreatectomy was performed and diffuse disease was identified. Sequence analysis also identified a previously reported (25) paternally inherited ABCC8 variant (p.Ala726Thr), but current evidence suggests that this variant is unlikely to be pathogenic.

Autosomal dominant inheritance of hyperinsulinism with a variable response to diazoxide has been reported (26,27,28,29,30,31). MacMullen et al (30) reported patients with dominantly inherited CHI with some affected parents carrying the same mutation whilst other parents were asymptomatic. In that study which included 17 diazoxide-unresponsive and 13 diazoxide-responsive patients, the mutation was maternally inherited in five patients from each group. In our study, two siblings with diazoxide-responsive HI were heterozygous for a novel ABCC8 missense mutation. Both children had inherited this mutation from their unaffected mother.

Preoperative diagnosis of focal HI is of great importance for determining the extent of pancreatectomy. 18F-DOPA PET CT scanning remains the only accurate method for localizing a focal lesion prior to surgery. Almost all patients with focal CHI have a paternally inherited recessively acting mutation in a KATP channel gene (17). In our series, two patients had a paternally inherited KATP channel gene mutation (ABCC8, n=1; KCNJ11, n=1). Since it is unavailable in our country, 18F-DOPA PET CT could not be performed in these patients. While CHI showed a spontaneous remission in the patient with KCNJ11 gene mutation at the age of 2.5 years, the other patient with an ABCC8 mutation was unresponsive to medical therapy and underwent near-total pancreatectomy.

The most common surgical method is near-total pancreatectomy in patients who are unresponsive to medical treatment. Post-pancreatectomy hyperglycemia may require either temporary or permanent insulin treatment. In long-term follow-up studies of patients with CHI who underwent pancreatectomy, diabetes has been reported in a limited number of patients (4,11,24,32,33). In 1984, Greene et al (4) reported five patients with CHI whose hypoglycemia could not be managed with medical therapy and who underwent pancreatectomy twice. Diabetes developed in all patients and insulin was required to achieve normoglycemia. Diabetes often occurs when patients undergo ≥95% pancreatectomy and then need a second operation (8,9,32,33). The interval between the surgery and the development of diabetes varies from immediately after (5,6,7,8,9,10,24) to several years after the (>18 years) surgery. Nevertheless, insulin-dependent diabetes frequently develops during adolescence (8,10,24,32). In one study, diabetes developed in 100% of patients by the 11th year following surgery (24). In the present study, diabetes developed immediately after the operation in one patient, and 2.5 years and 10 years after surgery in two patients. At their recent evaluation, all of these patients were on insulin treatment. The remaining four patients who underwent near-total pancreatectomy did not develop diabetes or had no evidence of exocrine pancreatic insufficiency during follow-up. However, this does not eliminate the risk of developing diabetes in the future. Therefore, these patients need to be evaluated periodically for diabetes.

It is not known whether there is a relation between diabetes development after pancreatectomy and the type of mutation causing CHI. It has been suggested that KATP channel gene mutations lead to an increase in the apoptosis of the beta cells of the pancreas (34). Leibowitz et al (35) demonstrated that insulin response to glucose stimulation was diminished in patients with CHI who underwent pancreatectomy. Impaired glucose tolerance and diabetes may present in these patients particularly during puberty when insulin response to hyperglycemia is blunted. However, ketoacidosis was not reported in the patients with diabetes, so one may consider that the residual pancreatic tissue secretes some insulin. The deterioration in glucose homeostasis is progressive in these patients and frank diabetes develops after many years.

Hypoglycemia requiring medical treatment in patients with CHI who underwent pancreatectomy may be explained by the regeneration of the residual pancreatic tissue (36). In our study group, all three patients who had diabetes subsequent to pancreatectomy had homozygous ABCC8 mutations. Two of the remaining four patients who underwent near-total pancreatectomy also had homozygous ABCC8 mutations and they still have no symptoms. The patient with no identifiable mutations and diffuse pancreatic disease and the patient with a paternally inherited ABCC8 mutation and a focal lesion have not yet developed diabetes. The limitation of our study is its retrospective design and having performed an 18F-DOPA PET-CT scan in only one patient prior to surgery.

Previous studies from our country regarding congenital hyperinsulinism did not report long-term follow-up data (37,38). Besides, in one of these reports, the diagnosis is based only on clinical and laboratory findings (37). In another multicenter study, clinical findings and genetic analyses of patients with CHI treated in four different centers were demonstrated, yet long-term follow-up was not reported (38).

Based on the information above, this present study stands out as the longest follow-up study of the patients with CHI from Turkey. Our study confirms that KATP channel gene mutations are the most common mutations causing CHI in Turkish patients. These mutations were identified in 85% of patients who underwent pancreatectomy. The development of diabetes mellitus after pancreatectomy may occur at any age, for that reason, patients should be screened regularly. We could not establish a relation between the type of mutation and the development of diabetes. Since 18F-DOPA PET-CT is not widely available, genetic analysis might be useful to identify focal CHI in those patients who cannot be subjected to 18F-DOPA PET-CT.

## Ethics

Ethics Committee Approval: Yes, Informed Consent: Genetic testing was performed by the University of Exeter Medical School with written informed consent obtained from parents of all patients.

Peer-review: External peer-reviewed.

## Figures and Tables

**Table 1:**
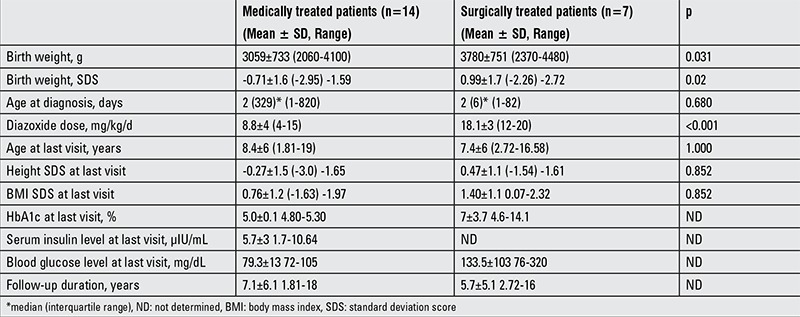
Clinical and biochemical characteristics of the medically and surgically treated patients

**Table 2:**
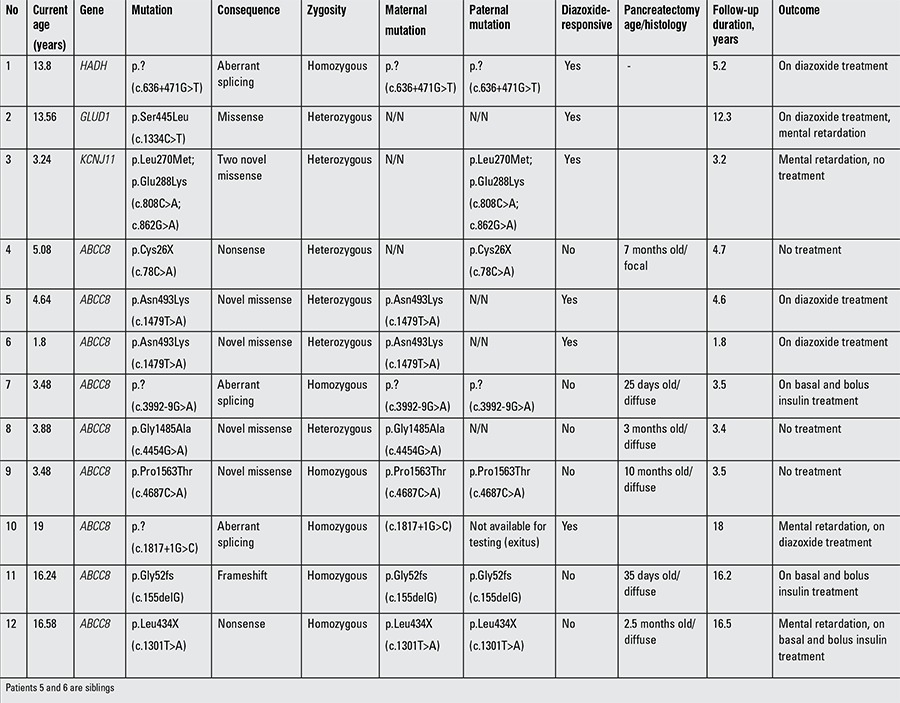
Genetic and clinical characteristics of 12 patients with mutation-positive congenital hyperinsulinism

## References

[1] Senniappan S, Shanti B, James C, Hussain K (2012). Hyperinsulinaemic hypoglycaemia: genetic mechanisms, diagnosis and management. J Inherit Metab Dis.

[2] Thornton PS, Satin-Smith MS, Herold K, Glaser B, Chiu KC, Nestorowicz A, Permutt MA, Baker L, Stanley CA (1998). Familial hyperinsulinism with apparent autosomal dominant inheritance: clinical and genetic differences from the autosomal recessive variant. J Pediatr.

[3] Verkarre V, Fournet JC, Lonlay P, Gross-Morand MS, Devillers M, Rahier J, Brunelle F, Robert JJ, Nihoul-Fékété C, Saudubray JM, Junien C (1998). Paternal mutation of the sulfonylurea receptor (SUR1) gene and maternal loss of 11p15 imprinted genes lead to persistent hyperinsulinism in focal adenomatous hyperplasia. J Clin Invest.

[4] Greene SA, Aynsley-Green A, Soltesz G, Baum JD (1984). Management of secondary diabetes mellitus after total pancreatectomy in infancy. Arch Dis Child.

[5] Shilyansky J, Fisher S, Cutz E, Perlman K, Filler RM (1997). Is 95% pancreatectomy the procedure of choice for treatment of persistent hyperinsulinemic hypoglycemia of the neonate?. J Pediatr Surg.

[6] Lovvorn HN, Nance ML, Ferry RJ, Stolte L, Baker L, O’Neill JA, Schnaufer L, Stanley CA, Adzick NS (1999). Congenital hyperinsulinism and the surgeon: lessons learned over 35 years. J Pediatr Surg.

[7] Lonlay-Debeney P, Poggi-Travert F, Fournet JC, Sempoux C, Dionisi Vici C, Brunelle F, Touati G, Rahier J, Junien C, Nihoul-Fékété C, Robert JJ, Saudubray JM (1999). Clinical features of 52 neonates with hyperinsulinism. N Eng J Med.

[8] Rother KI, Matsumoto JM, Rasmussen NH, Schwenk WF (2001). Subtotal pancreatectomy for hypoglycemia due to congenital hyperinsulinism: long-term follow-up of neurodevelopmental and pancreatic function. Pediatr Diabetes.

[9] McAndrew HF, Smith V, Spitz L (2003). Surgical complications of pancreatectomy for persistent hyperinsulinaemic hypoglycaemia of infancy. J Pediatr Surg.

[10] Jack MM, Greer RM, Thomsett MJ, Walker RM, Bell JR, Choong C, Cowley DM, Herington AC, Cotterill AM (2003). The outcome in Australian children with hyperinsulinism of infancy: early extensive surgery in severe cases lowers risk of diabetes. Clin Endocrinol (Oxf).

[11] Cherian MP, Abduljabbar MA (2005). Persistent hyperinsulinemic hypoglycemia of infancy (PHHI): Long-term outcome following 95% pancreatectomy. J Pediatr Endocrinol Metab.

[12] Koh TH, Aynsley-Green A, Tarbit M, Eyre JA (1988). Neural dysfunction during hypoglycaemia. Arch Dis Child.

[13] Kapoor RR, Flanagan SE, Fulton P, Chakrapani A, Chadefaux B, Ben-Omran T, Banerjee I, Shield JP, Ellard S, Hussain K (2009). Hyperinsulinism-hyperammonaemia syndrome: novel mutations in the GLUD1 gene and genotype-phenotype correlations. Eur J Endocrinol.

[14] Flanagan SE, Xie W, Caswell R, Damhuis A, Vianey-Saban C, Akcay T, Darendeliler F, Bas F, Guven A, Siklar Z, Ocal G, Berberoglu M, Murphy N, O’Sullivan M, Green A, Clayton PE, Banerjee I, Clayton PT, Hussain K, Weedon MN, Ellard S (;92:131-136.). Next generation sequencing reveals deep intronic cryptic ABCC8 and HADH splicing founder mutations causing hyperinsulinaemic hypoglycaemia by pseudoexon activation. Am J Hum Genet.

[15] Flanagan SE, Patch AM, Locke JM, Akcay T, Simsek E, Alaei M, Yekta Z, Desai M, Kapoor RR, Hussain K, Ellard S (2011). Genome-wide homozygosity analysis reveals HADH mutations as a common cause of diazoxide-responsive hyperinsulinemic-hypoglycemia in consanguineous pedigrees. J Clin Endocrinol Metab.

[16] Flanagan SE, Kapoor RR, Mali G, Cody D, Murphy N, Schwahn B, Siahanidou T, Banerjee I, Akcay T, Rubio-Cabezas O, Shield JP, Hussain K, Ellard S (2010). Diazoxide-responsive hyperinsulinemic hypoglycemia caused by HNF4A gene mutations. Eur J Endocrinol.

[17] Snider KE, Becker S, Boyajian L, Shyng SL, MacMullen C, Hughes N, Ganapathy K, Bhatti T, Stanley CA, Ganguly A (2013). Genotype and phenotype correlations in 417 children with congenital hyperinsulinism. J Clin Endocrinol Metab.

[18] Greer RM, Shah J, Jeske YW, Brown D, Walker RM, Cowley D, Bowling FG, Liaskou D, Harris M, Thomsett MJ, Choong C, Bell JR, Jack MM, Cotterill AM (2007). Genotype-phenotype associations in patients with severe hyperinsulinism of infancy. Pediatr Dev Pathol.

[19] Lonlay P, Fournet JC, Touati G, Groos MS, Martin D, Sevin C, Delagne V, Mayaud C, Chigot V, Sempoux C, Brusset MC, Laborde K, Bellane-Chantelot C, Vassault A, Rahier J, Junien C, Brunelle F, Nihoul-Fékété C, Saudubray JM, Robert JJ (2002). Heterogeneity of persistent hyperinsulinaemic hypoglycaemia. A series of 175 cases. Eur J Pediatr.

[20] Lord K, Dzata E, Snider KE, Gallagher PR, De León DD (2013). Clinical presentation and management of children with diffuse and focal hyperinsulinism: a review of 223 cases. J Clin Endocrinol Metab.

[21] Sogno Valin P, Proverbio MC, Diceglie C, Gessi A, di Candia S, Mariani B, Zamproni I, Mangano E, Asselta R, Battaglia C, Caruso-Nicoletti M, Mora S, Salvatoni A (2013). Genetic analysis of Italian patients with congenital hyperinsulinism of infancy. Horm Res Paediatr.

[22] Faletra F, Snider K, Shyng SL, Bruno I, Athanasakis E, Gasparini P, Dionisi-Vici C, Ventura A, Zhou Q, Stanley CA, Burlina A (2013). Co-inheritance of two ABCC8 mutations causing an unresponsive congenital hyperinsulinism: clinical and functional characterization of two novel ABCC8 mutations. gene.

[23] Thakur S, Flanagan SE, Ellard S, Verma IC (2011). Congenital hyperinsulinism caused by mutations in ABCC8 (SUR1) gene. Indian Pediatr.

[24] Arya VB, Senniappan S, Demirbilek H, Alam S, Flanagan SE, Ellard S, Hussain K (2014). Pancreatic endocrine and exocrine function in children following near-total pancreatectomy for diffuse congenital hyperinsulinism. PLoS One.

[25] Banerjee I, Skae M, Flanagan SE, Rigby L, Patel L, Didi M, Blair J, Ehtisham S, Ellard S, Cosgrove KE, Dunne MJ, Clayton PE (2011). The contribution of rapid KATP channel gene mutation analysis to the clinical management of children with congenital hyperinsulinism. Eur J Endocrinol.

[26] Thornton PS, MacMullen C, Ganguly A, Ruchelli E, Steinkrauss L, Crane A, Aguilar-Bryan L, Stanley CA (2003). Clinical and molecular characterization of a dominant form of congenital hyperinsulinism caused by a mutation in the high-affinity sulfonylurea receptor. Diabetes.

[27] Oçal G, Flanagan SE, Hacihamdioğlu B, Berberoğlu M, Siklar Z, Ellard S, Savas Erdeve S, Okulu E, Akin IM, Atasay B, Arsan S, Yağmurlu A (2011). Clinical characteristics of recessive and dominant congenital hyperinsulinism due to mutation(s) in the ABCC8/KCNJ11 genes encoding the ATP-sensitive potasium channel in the pancreatic beta cell. J Pediatr Endocrinol Metabol.

[28] Pinney SE, MacMullen C, Becker S, Lin YW, Hanna C, Thornton P, Ganguly A, Shyng SL, Stanley CA (2008). Clinical characteristics and biochemical mechanisms of congenital hyperinsulinism associated with dominant KATP channel mutations. J Clin Invest.

[29] Pinney SE, Ganapathy K, Bradfield J, Stokes D, Sasson A, Mackiewicz K, Boodhansingh K, Hughes N, Becker S, Givler S, Macmullen C, Monos D, Ganguly A, Hakonarson H, Stanley CA (2013). Dominant form of congenital hyperinsulinism maps to HK1 region 10q. Horm Res Paediatr.

[30] MacMullen CM, Zhou Q, Snider KE, Tewson PH, Becker SA, Aziz AR, Ganguly A, Shyng SL, Stanley CA (2011). Diazoxide-unresponsive congenital hyperinsulinism in children with dominant mutations of the β-cell sulfonylurea receptor SUR1. Diabetes.

[31] Flanagan SE, Kapoor RR, Banerjee I, Hall C, Smith VV, Hussain K, Ellard S (2011). Dominantly acting ABCC8 mutations in patients with medically unresponsive hyperinsulinaemic hypoglycaemia. Clin Genet.

[32] Meissner T, Wendel U, Burgard P, Schaetzle S, Mayatepek E (2003). Long-term follow-up of 114 patients with congenital hyperinsulinism. Eur J Endocrinol.

[33] Tyrrell VJ, Ambler GR, Yeow WH, Cowell CT, Silink M (2001). Ten years’ experience of persistent hyperinsulinaemic hypoglycemia of infancy. J Paediatr Child Health.

[34] Mazor-Aronovitch K, Landau H, Gillis D (2009). Surgical versus non-surgical treatment of congenital hyperinsulinism. Pediatr Endocrinol Rev.

[35] Leibowitz G, Glaser B, Higazi AA, Salameh M, Cerasi E, Landau H (1995). Hyperinsulinemic hypoglycemia of infancy (nesidioblastosis) in clinical remission: high incidence of diabetes mellitus and persistent beta-cell dysfunction at long-term follow-up. J Clin Endocrinol Metab.

[36] Schönau E, Deeg KH, Huemmer HP, Akcetin YZ, Böhles HJ (1991). Pancreatic growth and function following surgical treatment of nesidioblastosis in infancy. Eur J Pediatr.

[37] Ağladıoğlu SY, Savaş Erdeve S, Cetinkaya S, Baş VN, Peltek Kendirci HN, Onder A, Aycan Z (2013). Hyperinsulinemic hypoglycemia: experience in a series of 17 cases. J Clin Res Pediatr Endocrinol.

[38] Demirbilek H, Arya VB, Ozbek MN, Akinci A, Dogan M, Demirel F, Houghton J, Kaba S, Guzel F, Baran RT, Unal S, Tekkes S, Flanagan SE, Ellard S, Hussain K (2014). Clinical characteristics and phenotype-genotype analysis in Turkish patients with congenital hyperinsulinism; predominance of recessive KATP channel mutations. Eur J Endocrinol.

